# Minimally Invasive Access in Endodontics: A National Survey of Brazilian Specialists

**DOI:** 10.1590/0103-644020266955

**Published:** 2026-08-24

**Authors:** Fabienne de Freitas Rodrigues Moraes, Roberta Fonseca de Castro, Emmanuel João Nogueira Leal da Silva, Francisco Montagner, Juliana Melo da Silva Brandão

**Affiliations:** 1 Department of Endodontics, Pará Federal University, Belém, Pará, Brazil; 2 Department of Endodontics, Rio de Janeiro State University, Rio de Janeiro, RJ, Brazil; 3 Department of Endodontics, Grande Rio University, Duque de Caxias, RJ, Brazil; 4 Department of Conservative Dentistry, Federal University of Rio Grande do Sul, Porto Alegre, Brazil

**Keywords:** Endodontics, minimally invasive surgical procedures, professional practice, evidence-based practice, perception

## Abstract

This study aims to evaluate Brazilian endodontists’ perceptions of minimally invasive access cavities, characterize access-design choices, and identify the information sources informing these decisions. This cross-sectional survey randomly sampled endodontists registered with the Federal Council of Dentistry in 2021. Consenting participants completed a previously validated questionnaire. A minimum sample of 376 was calculated (assumed proportion 50%; margin of error 5%). Descriptive statistics summarized responses, and Pearson’s chi-square tested associations between access type and covariates (α = 0.05). Of 378 respondents, most reported using traditional access for anterior (55.8%) and posterior teeth (66.4%). On a 0-5 scale, the modal self-rated knowledge of minimally invasive access cavities was 4 (28%). Most agreed/strongly agreed that minimally invasive access cavities: increases fracture resistance (49.5%); hinders canal location (84.4%); impairs instrument centering during preparation (73.5%); raises the risk of apical transportation (43.4%) and iatrogenic deviations (63.0%); contributes to debris accumulation (60.1%); compromises pulp-chamber cleaning (74.6%); elevates the likelihood of instrument fracture (68.3%); and increases the risk of coronal discoloration (57.6%). Scientific articles were the most frequently cited source of professional updating (44.2%). Brazilian endodontists perceived multiple procedural risks associated with minimally invasive access cavities and often believed it improves fracture resistance, a view not supported by current evidence. These findings reveal a perception-evidence gap and underscore the need to strengthen critical appraisal and evidence integration in education and clinical decision-making on access cavity design.

## Resumo

Este estudo tem como objetivo avaliar as percepções dos endodontistas brasileiros sobre as cavidades de acesso minimamente invasivas, caracterizar as escolhas da modalidade de acesso e identificar as fontes de informação que embasam essas decisões. Este estudo transversal teve como amostra aleatória endodontistas registrados no Conselho Federal de Odontologia em 2021. Os participantes que consentiram em participar, preencheram a um questionário previamente validado. Uma amostra mínima de 376 foi calculada (proporção assumida de 50%; margem de erro de 5%). As respostas foram sumarizadas por estatísticas descritivas e o teste do qui-quadrado de Pearson testou as associações entre o tipo de acesso e as covariáveis ​​(α = 0,05). Dos 378 entrevistados, a maioria relatou usar cavidades de acesso tradicional para dentes anteriores (55,8%) e posteriores (66,4%), Em uma escala de 0 a 5, o conhecimento autoavaliado sobre cavidades de acesso minimamente invasivas foi 4 (28%). A maioria concordou/concordou totalmente que cavidades de acesso minimamente invasivas: aumentam a resistência à fratura (49,5%); dificultam a localização do canal (84,4%); prejudicam a centralização do instrumento durante o preparo (73,5%); aumentam o risco de transporte apical (43,4%) e desvios iatrogênicos (63,0%); contribuem para o acúmulo de detritos (60,1%); comprometem a limpeza da câmara pulpar (74,6%); aumentam a probabilidade de fratura do instrumento (68,3%); e elevam o risco de descoloração coronária (57,6%). Artigos científicos foram a fonte de atualização profissional mais citada (44,2%). Endodontistas brasileiros percebem múltiplos riscos processuais associados a cavidades de acesso minimamente invasivas e frequentemente acreditam que acessos minimamente invasivos melhoram a resistência à fratura, uma visão não apoiada por evidências atuais. Essas descobertas revelam uma lacuna entre percepção e evidência e ressaltam a necessidade de fortalecer a avaliação crítica e a integração de evidências na educação e na tomada de decisão clínica sobre o design de cavidades de acesso.

**Figure:**
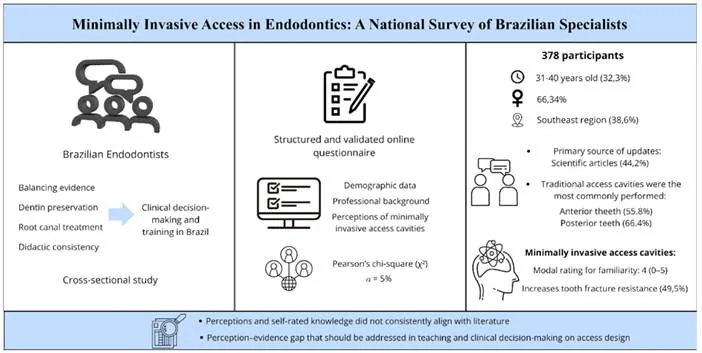

## Introduction

The concept of minimally invasive access cavities was introduced by Clark and Khademi^1^, in opinion-based articles. The authors hypothesized that retaining as much of the pulp chamber roof as possible would preserve pericervical dentine, thereby helping distribute occlusal forces along the tooth's long axis and improving the fracture resistance of endodontically treated teeth. Since then, minimally invasive access cavity design has been investigated across multiple aspects of endodontic therapy, including fracture resistance^2^ ^,^ ^3^ ^,^ ^4^ ^,^ ^5^ ^,^ ^6^ ^,^ ^7^ ^,^ ^8^ ^,^ ^9^ ^,^ ^10^, canal location^3^ ^,^ ^11^, potential for iatrogenic deviations and apical transportation^3^ ^,^ ^7^ ^,^ ^12^, instrument fracture^9^ ^,^ ^13^, irrigation efficiency^6^ ^,^ ^14^, pulp chamber cleaning, and coronal discoloration^7^ ^,^ ^8^.

Despite extensive investigation, definitive clinical advantages of minimally invasive access cavities over traditional access designs remain unproven. In fact, the current evidence base consists primarily of laboratory-based studies that, taken together, do not support routine clinical adoption. Nevertheless, some clinicians and opinion leaders continue to promote minimally invasive access cavities, particularly on social media, creating visibility that can outpace the evidence. This tension is consequential in educational settings, where consistency, reproducibility, and didactic clarity are essential^15^ ^,^ ^16^. Collectively, these factors motivate an examination of how endodontists perceive and apply minimally invasive access cavities in clinical practice.

Beyond published evidence and procedural performance, treatment planning is shaped by clinicians’ risk-benefit appraisals, case selection, and the perceived trade-offs between dentin preservation and canal identification/chemomechanical debridement. In practice, clinical norms diffuse through training histories, institutional protocols, referral networks, and continuing education, collectively shaping how techniques enter routine care^17^. Understanding how endodontists view minimally invasive access cavity preparation can clarify current patterns of use, highlight perceived barriers and facilitators, and inform both educational curricula (where consistency and didactic clarity are essential) and restorative coordination, ultimately guiding evidence-aligned adoption.

The increasing variety of access cavity designs and the wide range of abbreviations used in the literature have resulted in overlapping and inconsistent terminology^16^ ^,^ ^18^. To promote clarity in the understanding of endodontic access types and their corresponding terminology, this study utilized standardized images based on the classification proposed by Silva et al. (2020)^18^, which includes: traditional access cavity (TradAC), conservative access cavity (ConsAC), ultra-conservative access cavity (UltraAC), conservative access cavity with divergent walls (ConsAC.DW), ultra-conservative access cavity performed at the incisal edge (UltraAC.Inc), and directed access cavity (TrussAC).

To clarify how these dynamics-balancing evidence, dentin preservation, root canal treatment, and didactic consistency-translate into clinical decision-making and training in Brazil, this cross-sectional investigation used a structured, validated questionnaire to (i) assess Brazilian endodontists’ perceptions of minimally invasive access cavities, (ii) identify their access-cavity design preferences, (iii) determine the sources of information that underpin these choices, and (iv) evaluate how these perspectives correspond to current approaches to minimally invasive access cavities preparation in educational contexts.

## Materials and methods

### Study Design

This cross-sectional study was conducted in accordance with the STROBE (Strengthening the Reporting of Observational Studies in Epidemiology) guidelines^19^. Brazilian endodontists registered with the Federal Dental Council were recruited between August 2021 and January 2022 through direct contact, email invitations, and social media outreach. Data were collected using a semi-structured electronic questionnaire, specifically developed for this study and hosted on Google Forms.

### Ethical and Legal Aspects

This study was approved by the Local Research Ethics Committee (Protocol No. 44694221.6.0000.0018). The research protocol adhered to the ethical principles outlined in Brazilian National Health Council Resolutions 466/2012 and 510/2016, as well as the guidelines for conducting research in virtual environments, as recommended by the National Research Ethics Commission (CONEP) through Circular Letter No. 2/2021/CONEP/SECNS/MS. All participants provided electronic informed consent prior to enrollment, confirming their voluntary participation in the study.

### Questionnaire Development and Validation

The questionnaire was developed and validated based on the methodology proposed by Magno et al. (2019)^20^. The process comprised four sequential stages: (a) initial development of the questionnaire; (b) review by a panel of 10 practicing endodontists; (c) review by 10 academic endodontists; and (d) refinement and finalization of the instrument.

The final instrument had three sections: demographics, professional background, and perceptions of minimally invasive access cavities. A systematic review informed perception items of the literature. Likert-type scales captured the reported frequency of use for each access design and respondents' self-reported knowledge of minimally invasive approaches. Opinion items were rated on a five-point agreement scale (strongly agree, agree, neutral, disagree, strongly disagree)^21^.

### Sample Size

The minimum sample size was estimated in G*Power 3.1 software, using the 2021 roster of registered endodontists in Brazil (N = 16,658; Federal Dental Council). Assuming a conservative 50% response distribution and a 5% margin of error, the required sample was 376 participants ^22^. Eligible endodontists were randomly selected from the registry to reach this target.

### Statistical Analysis

Data were entered into Microsoft Excel 2016 and analyzed as follows. Categorical variables were summarized as absolute frequencies and percentages. Age and years of professional experience were captured as open-ended fields and subsequently grouped into categories for analysis. Associations between the type of endodontic access performed and covariates of interest were tested using Pearson's chi-square (χ²). All statistical analyses were conducted using Jamovi (version 1.6), with a significance level of 5%.

## Results

**Figure 1:**
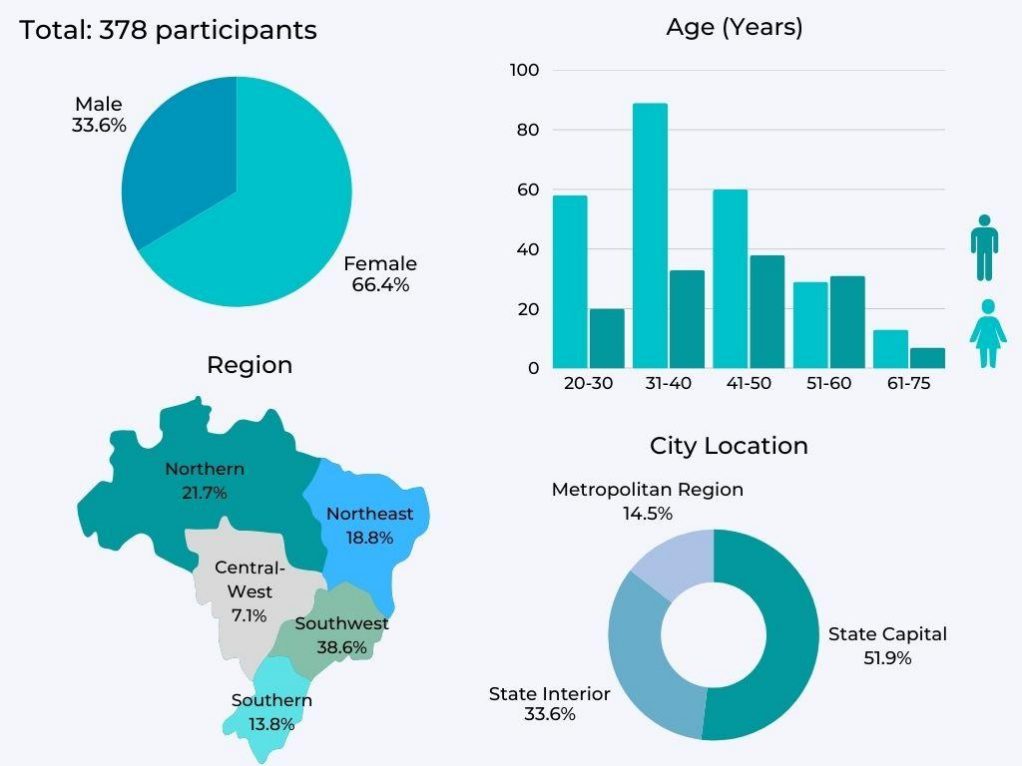
Demographic characteristics of respondents, including gender, age group, and geographic region.

Of 379 responses received, one respondent declined consent; 378 questionnaires were analyzed. Most participants were 31-40 years old (122/378; 32.3%), female (251/378; 66.4%), residing in the Southeast region (146/378; 38.6%), and practicing in state capitals (196/378; 51.9%) (Figure 1).

Most respondents had 1-10 years of endodontic practice (193/378; 51.1%). About one-third reported an Endodontics specialization as their sole postgraduate credential (128/378; 33.9%); among those with additional training, a stricto sensu master’s degree was most common (97/378; 25.7%). Scientific articles were cited as the primary source of professional updates (167/378; 44.2%).

**Figure 2:**
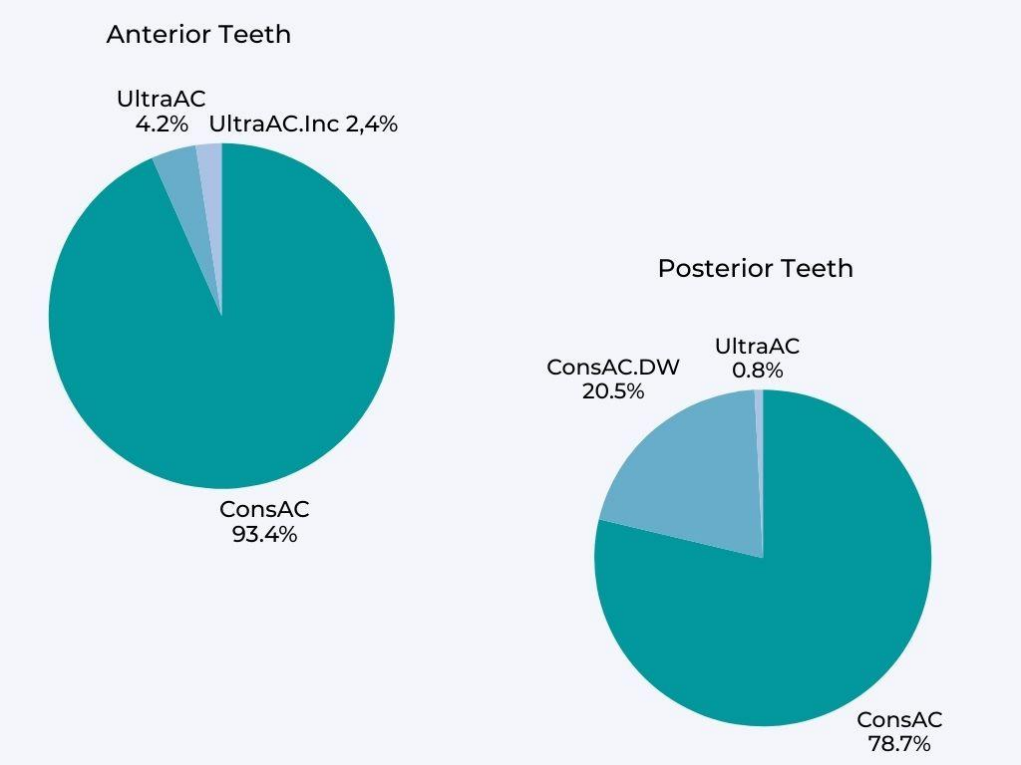
Frequency of minimally invasive access cavity designs performed in anterior and posterior teeth. ConsAC: conservative access cavity; ConsAC.DW: conservative access cavity with divergent walls; UltraAC: ultra-conservative access cavity; UltraAC.Inc: ultra-conservative access cavity performed at the incisal edge.

Traditional access cavities (TradAC) were the most performed in routine care for both anterior (55.8%) and posterior teeth (66.4%). Among minimally invasive designs (ConsAC, UltraAC, ConsAC.DW, UltraAC.Inc, TrussAC), Conservative Access (ConsAC) was the most widely used, reported by 93.4% of respondents for anterior teeth and 78.7% for posterior teeth (Figure 2).

On a 5-point scale (1 = never; 5 = always), anterior teeth showed a modal response of 5 for TradAC (43.9%) and 4 for ConsAC (26.7%). UltraAC and UltraAC.Inc were seldom used, with 81% and 76.2% reporting 1 (never), respectively. For posterior teeth, the modal response was 5 for TradAC (52.4%), while 1 (never) was the most frequent category for ConsAC (29.4%), ConsAC.DW (55%), UltraAC (86.2%), and TrussAC (91%).

On a 0-5 self-rating scale (0 = no knowledge; 5 = high knowledge), the modal rating for familiarity with minimally invasive access cavity was 4 (28%). Respondents who rated 4 most commonly had 6-10 years of professional experience (24.5%), and there was a significant association between knowledge rating and years of experience (χ², p < 0.001). When asked which sources supported replacing TradAC with minimally invasive access cavity designs, the most frequently cited resource was scientific articles (24%).

**Figure 3:**
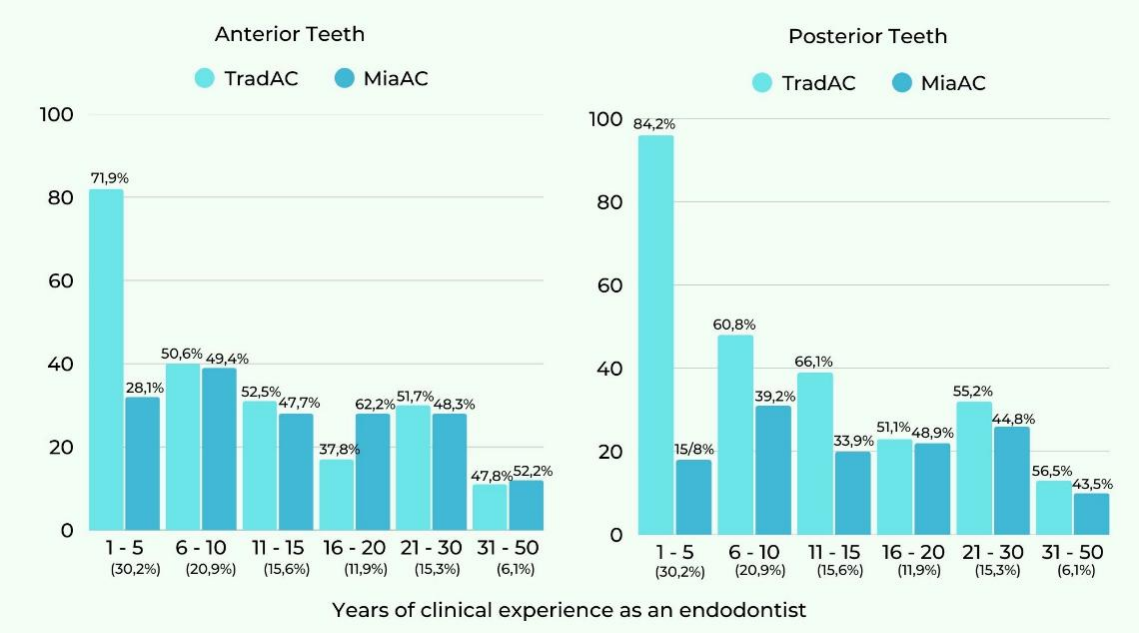
Association between the type of endodontic access performed in anterior (p = 0.001) and posterior (p < 0.001) teeth and years of clinical experience as an endodontist. TradAC - traditional access cavity; MiaAC - minimally invasive access cavities. Chi-square test.

Stratifying anterior-tooth access by professional characteristics, endodontists who reported using minimally invasive access cavities were most commonly those with 6-10 years of experience (23.4%; χ², p = 0.001; Figure 3). They also most frequently cited scientific articles as their primary source of continuing education (39.5%; χ², p = 0.007; Table 1). Among respondents who reported using minimally invasive access cavities for posterior teeth, the most common profile included 6-10 years of endodontic experience (24.4%; χ², p < 0.001; Figure 3), a master’s degree as additional academic training (33.9%; χ², p = 0.009), scientific articles as the main source of continuing education (43.3%; χ², p = 0.018), and self-rated knowledge = 4 on a 0-5 scale (33.1%; χ², p < 0.001) (Table 2).

**Table:** 

	n (%)			p-value
	TradAc	MiaAc	Total	
In-person refresher and improvement courses	29 (13.7)	34 (20.4)	63 (16.7)	0.007*
Online refresher and improvement courses	27 (12.8)	25 (15.0)	52 (13.8)	
Congresses	13 (6.2)	25 (15.0)	38 (10.1)	
Books	6 (2.8)	1 (0.6)	7 (1.9)	
Scientific articles	101 (47.9)	66 (39.5)	167 (44.2)	
Talking to more experienced colleagues	11 (5.2)	2 (1.2)	13 (3.4)	
Social media	16 (7.6)	10 (6.0)	26 (6.9)	
Others	0 (0.0)	1 (0.6)	1 (0.3)	
I have not been keeping up to date with Endodontics	8 (3.8)	3 (1.8)	11 (2.9)	
Total	211 (55.8)	167 (44.2)	378 (100)	

Traditional access cavity (TradAc) and Minimally invasive access cavities (MiaAc)

*Chi-square test

Most respondents agreed/strongly agreed that minimally invasive access cavities: (i) increases tooth fracture resistance (49.5%); (ii) hinders locating canal orifices (84.4%); (iii) impairs instrument centering during chemomechanical preparation (73.5%); (iv) increases the likelihood of apical transportation (43.4%); (v) compromises irrigation efficiency, leading to greater debris accumulation within the root canal system (60.1%); (vi) impairs pulp-chamber cleaning after obturation (74.6%); (vii) raises the risk of iatrogenic deviations during instrumentation (63.0%); (viii) increases the potential for instrument fracture (68.3%); and (ix) heightens the risk of tooth discoloration (57.6%). Additionally, 74.1% agreed that minimally invasive access cavities result in greater retention of pulp-tissue remnants within the chamber. Notably, 68.3% also agreed that, with adequate magnification, illumination, and the use of ultrasonic tips, the access design does not affect the ability to locate root canals.

**Table 2:** Association between the type of endodontic access performed in posterior teeth and professional characteristics, including additional academic training, source of continuing education in Endodontics, and self-reported level of knowledge regarding minimally invasive access cavities.

	n (%)			p-value
	TradAc	MiaAc	Total	
Additional training				0.009*
Only specialization in Endodontics	92 (36,7)	36 (28,3)	128 (33,9)	
Yes, updates	27 (10,8)	6 (4,7)	33 (8,7)	
Yes, specialization in other areas	36 (14,3)	14 (11,0)	50 (13,2)	
Yes, residency program	4 (1,6)	0 (0,0)	4 (1,1)	
Yes, master's degree	54 (21,5)	43 (33,9)	97 (25,7)	
Yes, doctorate	31 (12,4)	26 (20,5)	57 (15,1)	
Yes, postdoctoral	7 (2,8)	2 (1,6)	9 (2,4)	
Source for Endodontics Updates				0.018*
In-person refresher and improvement courses	38 (15,1)	25 (19,7)	63 (16,7)	
Online refresher and improvement courses	37 (14,7)	15 (11,8)	52 (13,8)	
Congresses	17 (6,8)	21 (16,5)	38 (10,1)	
Books	6 (2,4)	1 (0,8)	7 (1,9)	
Scientific articles	112 (44,6)	55 (43,3)	167 (44,2)	
Talking to more experienced colleagues	10 (4,0)	3 (2,4)	13 (3,4)	
Social media	22 (8,8)	4 (3,1)	26 (6,9)	
Others	0 (0,0)	1 (0,8)	1 (0,3)	
I have not been keeping up to date with Endodontics	9 (3,6)	2 (1,6)	11 (2,9)	

Traditional access cavity (TradAc) and Minimally invasive access cavities (MiaAc)

*Chi-square test

## Discussion

From a dental-education perspective, minimally invasive access cavities have often been encountered first outside formal programs, leaving variability in how-and whether-it is critically appraised and taught. Understanding how practicing endodontists perceive minimally invasive access cavities, which designs they prefer, and where they obtain supporting information is essential to align curricula with evidence, calibrate faculty, and set competency expectations for case selection and adjunctive technologies (e.g., magnification, ultrasonics). To inform these decisions, a national cross-sectional survey was conducted using a structured, validated questionnaire to assess perceptions, map information sources, and examine concordance with the current literature.

A central finding was that 49.5% of respondents believed that minimally invasive access cavities increase tooth fracture resistance-an expectation not consistently supported by current in vivo laboratory evidence when minimally invasive access cavities are compared with traditional access designs ^3^ ^,^ ^4^ ^,^ ^5^ ^,^ ^6^ ^,^ ^7^ ^,^ ^8^ ^,^ ^9^ ^,^ ^10^ ^,^ ^16^ ^,^ ^23^ ^,^ ^24^ ^,^ ^25^ ^,^ ^26^. In contrast, perceptions regarding procedural impacts largely aligned with the literature: most respondents agreed that minimally invasive access cavities can hinder canal orifice location ^3^ ^,^ ^11^, impair instrument centering ^12^, raise the risk of apical transportation and iatrogenic deviations ^3^ ^,^ ^7^ ^,^ ^12^, and compromise irrigation/cleaning with greater debris accumulation ^14^ ^,^ ^16^. They also associated minimally invasive access cavities with more residual material in the chamber and a higher likelihood of coronal discoloration ^7^ ^,^ ^8^ and with a greater risk of instrument fracture ^9^ ^,^ ^13^.

Use patterns reflect this caution in certain ways. TradAC remained the most common approach for both anterior and posterior teeth, while UltraAC, UltraAC.Inc, and TrussAC were infrequently selected. Notably, 68.3% agreed that with adequate magnification, illumination, and ultrasonic tips, access design does not affect canal location, suggesting that operator aids may mitigate some drawbacks. In fact, evidence shows that the design of the access cavity does not significantly affect canal detection when magnification associated with ultrasonic tips is used ^3^ ^,^ ^11^ However, technology uptake is uneven: while reports from the United States note substantial microscope adoption ^(^ ^27^ ^,^ ^28^, Brazilian data show more limited use ^29^, raising concerns about adopting minimally invasive access cavities without consistent access to adjuncts needed for safe execution.

Information sources may help explain perception-evidence gaps. Although scientific articles were frequently cited for professional updating, more than half of respondents also relied on secondary interpretations (courses, congresses, peer exchange, social media), which can amplify enthusiasm ahead of high-level evidence ^30^. Evidence-based practice requires integrating research, clinical expertise, and patient preferences ^31^. However, many clinicians equate evidence-based practice with expert opinion, potentially reinforcing early adoption of appealing concepts despite limited clinical data.

Professional correlates of minimally invasive access cavity use were noteworthy: among those reporting minimally invasive access cavities in posterior teeth, the most common profile included 6-10 years of experience, master 's-level training, reliance on scientific articles, and self-rated knowledge of 4/5. Paradoxically, this group's confidence and its use of literature coexisted with perceptions that are not consistently corroborated by current evidence. Possible explanations include case-selection heuristics, perceived restorative advantages (e.g., dentin preservation for ferrule), availability of magnification/ultrasonics, and diffusion of norms through continuing education and referral networks rather than through controlled clinical data.

It is important to emphasize that this study did not address the clinical outcomes of endodontists or decision-making in more complex cases, such as those with complex root anatomy or significant coronal loss. However, the evidence found demystifies the knowledge base and judgment that underpin routine practices and clinical decision-making.

Educational implications are context-dependent. International surveys report increasing incorporation of contemporary endodontic technologies into dental-school curricula ^32^, whereas Brazilian programs may face resource constraints that limit consistent access to magnification and ultrasonics ^33^. In this setting, curricula should (i) delineate the current limits of clinical evidence for minimally invasive access cavities; (ii) explicitly teach procedural trade-offs-effects on canal negotiation, debridement, and irrigant dynamics; and (iii) define competencies in adjuncts (magnification, illumination, ultrasonics) and case selection to minimize iatrogenic events. Until robust clinical trials demonstrate patient-centered benefits, routine adoption of minimally invasive access cavities is not advisable; when contemplated, it should be limited to carefully selected cases with explicit indications, appropriate adjuncts, and documented safeguards ^34^.

This study has limitations inherent to web-based surveys, including potential selection and response biases, as well as the possibility of misinterpretation. Nevertheless, rigorous validation procedures and incorporated illustrative images of access types were implemented to maximize clarity. In this context, the observation that most endodontists agreed that minimally invasive access cavities increase tooth fracture resistance-despite the absence of consistent supporting evidence-suggests that clinical preferences may be shaped more by prevailing trends and the appeal of conservative concepts than by robust data. The widespread perception of minimally invasive access cavities as consonant with contemporary, minimally invasive medical practice^16^ may further promote their adoption based on intuition or conceptual appeal rather than critical appraisal of the available evidence. Together, these findings point to a gap between perceived and evidence-based benefits in clinical decision-making.

## Conclusion

Brazilian endodontists commonly perceived that minimally invasive access cavities increase fracture resistance, a view not supported by current evidence. Although many cited scientific articles as their main learning source, perceptions and self-rated knowledge did not consistently align with the literature. These findings highlight a perception-evidence gap that should be addressed in teaching and clinical decision-making on access design.

## Data Availability

The research data are available within the article
